# *Mansonia africana* and *Mansonia uniformis* are Vectors in the transmission of *Wuchereria bancrofti* lymphatic filariasis in Ghana

**DOI:** 10.1186/1756-3305-5-89

**Published:** 2012-05-07

**Authors:** Josephine Ughasi, Hilaria Esiawonam Bekard, Maimouna Coulibaly, Delphina Adabie-Gomez, John Gyapong, Maxwell Appawu, Michael David Wilson, Daniel Adjei Boakye

**Affiliations:** 1 Department of Parasitology, Noguchi Memorial Institute for Medical Research, University of Ghana, P.O. Box LG 581, Legon-Accra, Ghana; 2 The Malaria Research and Training Centre, Bamako, Mali; 3 African Regional Programme for Insect Science (ARPPIS), University of Ghana, Legon, Ghana; 4 Lymphatic Filariasis Support Centre for Africa, Noguchi Memorial Institute for Medical Research, University of Ghana, P.O. Box LG 581, Legon-Accra, Ghana

**Keywords:** *Mansonia* species, *Wuchereria bancrofti*, Vectors, Lymphatic filariasis, Ghana

## Abstract

**Background:**

Recent data from Ghana indicates that after seven rounds of annual mass drug administration (MDA) there is still sustained transmission albeit at low levels in certain areas where *Anopheles melas*, *An. gambiae* s.s., *Mansonia* and *Culex* species are the main biting mosquitoes. *Anopheles gambiae* s.l. and *An. funestus* are the known vectors in Ghana and a recent report indicated that *An. melas* could transmit at low level microfilaraemia. However, because *An. melas* is not found everywhere there was the need to determine whether any of the other culicine species could also be playing a role in the transmission of LF.

**Methods:**

Indoor mosquitoes collected once a month for three months using pyrethrum spray catches in six communities within the Kommenda-Edina-Eguafo-Abirem (KEEA) District, Central Region of Ghana were morphologically identified, dissected and examined for the presence of *W. bancrofti*. Additionally, stored mosquito samples collected during previous years in 8 communities from the Gomoa District also in the Central Region were similarly processed. The identities of all *W. bancrofti* parasites found were confirmed using an established PCR method.

**Results:**

A total of 825 indoor resting mosquitoes comprising of 501 *Anopheles* species, 239 *Mansonia* species, 84 *Culex* species and 1 *Aedes* species were dissected and examined for the presence of *W. bancrofti*. *Mansonia africana* had infection and infectivity rates of 2.5*%*. and 2.1% respectively. *Anopheles gambiae* s.l. had an infection rate of 0.4% and a similar infectivity rate. None of the *Culex* sp. and *Aedes* sp were found with infection. From the stored mosquitoes the infection and infectivity rates for *M. africana* were 7.6% (N = 144) and 2.8% respectively whilst the corresponding rates for *M. uniformis* were 2.9% (N = 244) and 0.8%.

**Conclusions:**

This is the first report of *Mansonia* species as vectors of lymphatic filariasis (LF) in Ghana and in West Africa since that of 1958 in Guinea. The revelation of a hitherto unrecognised vector which is possibly more efficient in transmission than the recognised ones has a profound implication for elimination of lymphatic filariasis programmes in the sub-region.

## Background

*Wuchereria bancrofti,* the causative agent of lymphatic filariasis (LF) is transmitted by mosquito species belonging to *Anopheles, Aedes, Culex and Mansonia* depending on the geographic area. In East Africa, *Anopheles* and *Culex* species are known vectors but in West Africa only *Anopheles* species are the reported vectors of the disease. In Ghana *An. gambiae* s.l and *An. funestus* are the main vectors [1,2] with *An. pharoensis* playing a minor role [3]. Several previous studies have determined that *Culex* species are refractory to *W. bancrofti* in West Africa [4,5] and Ghana [6]. Although *Mansonia* species are identified among biting mosquito populations in LF endemic areas of Ghana their role in LF transmission are as yet unknown and only one report of vectorial status in West Africa has been published [7].

The Global Programme to Eliminate Lymphatic Filariasis (GPELF) strategy is mass drug administration to entire endemic communities. This strategy is based on the assumption that when circulating microfilariae (mf) levels are reduced below a certain threshold that decreases availability of mf to vectors, the transmission of *W. bancrofti* will be interrupted, especially if the vectors are anophelines [8]. This reasoning is based on the observation that anophelines exhibit the process of ‘facilitation’ whereby the number of ingested mfs developing to infective stages (L3) increases with increasing number of ingested mfs. The opposite, which is the process of ‘limitation’ is observed in culicines [9,10]. Thus the successful elimination of LF by MDA is considered to be achievable in areas with *Anopheles-W. bancrofti* relationships compared to *Culex* or *Aedes* or *Mansonia* – *W. bancrofti* combinations [11]. This indicates that an understanding of the local vector-parasite combination is critical for the elimination of LF.

Since *Anopheles* species are the known vectors in Ghana it was assumed that after 5 – 6 rounds of once a year of MDA with a combination of ivermectin and albendazole it should be possible to interrupt transmission of LF [8,12]. Recent evaluations of the impact of 6 rounds of MDA in Ghana have revealed residual transmission is still ongoing in some communities (Dr Nana Biritwum unpublished data). Collections of mosquitoes in these LF endemic areas have shown large numbers of *Culex* and *Mansonia* species and while it has been established that *Culex* does not transmit the disease in these areas [6], the status of *Mansonia* is not known. We therefore conducted an initial investigation regarding the involvement of *Mansonia* species in LF transmission in communities in one of these endemic areas i.e. Komenda-Edina-Eguafo-Abirem (KEEA) District. When infective mosquitoes were found by this initial study we then revisited stored *Mansonia* mosquitoes which had been collected during previous entomology studies at another endemic district i.e. Gomoa District and found all the developmental stages of *W. bancrofti* in *Mansonia* species. We report in this article the results obtained and discuss the implications for LF elimination in Ghana.

## Methods

### Description of study sites

#### KEEA District

The district is located in the Central Region of Ghana between longitude 1°20’ West and 1°40’ West, and latitude 5°05’ North and 5°15’ North. There had been 5 MDAs in this area by 2008 and a parasitological survey carried out in the study communities found seven people positive for *W. bancrofti* out of a total of 799 screened (0.88%). The number of microfilariae per 100 μl of blood ranged from 3 to 279 using the counting chamber method. The mosquito species found in this area are *An. gambiae* s.l., *An. funestus*, *An*. *pharoensis, Aedes* species, *Culex* species and *Mansonia* species.

## Gomoa District

The Gomoa district is also located in the Central Region and found between longitudes 0°22’ West and 0°54’ West, and latitudes 5°14’ North and 5°35’ North. There has been 7 MDA’s in this area and the last microfilariaemia prevalence was 0.4%. *Anopheles gambiae* s.l, *An. funestus, An. pharoensis*, *Culex* species, *Aedes* species and *Mansonia* species are the known biting mosquitoes in the district.

## Field sampling of mosquitoes

### KEEA collections

Indoor resting adult mosquitoes were collected from randomly selected compounds in six villages Sanka, Epoano, Atabadze, Anyinase, Ponkrom, Bandor in the KEEA District. The collections were done on two consecutive days in each month between 0500 H and 0800 H by a team of eight collectors during August, October and December 2009 using PSC method. All knocked-down mosquitoes were picked and placed on moist filter paper lining appropriately labelled Petri dishes and transported to the laboratory for processing.

## Gomoa collections

The stored mosquitoes used were collected between, 2000 – 2003 and were part of those obtained from July to December each year during entomological studies aimed at monitoring and evaluating MDA activities from 2000 – 2008. These were mosquitoes caught indoors using the HLC method simultaneously in all eight communities from 1800 H to 0600 H each day for four consecutive days for each of the six months. The mosquitoes collected at the time were kept in paper cups, transported to the laboratory, killed and sorted out according to genus to separate *Anopheles* species from the rest, including *Mansonia* spp, which were stored over silica gel until they were used for this study.

## Processing of mosquitoes

Each mosquito was identified using the morphological keys of Gillies and De Meillon [13] and Gillies and Coetzee [14] and sorted out into the different species of *AedesAnophelesCulex* and *Mansonia*.

All mosquitoes collected from KEEA were processed for *W. bancrofti* infections, while only the stored *Mansonia* spp from Gomoa District were processed for this study, because the *Anopheles* and *Culex* species obtained there had been used by earlier studies [Boakye DA, unpublished data].

## Mosquito dissections

The head, thorax and abdomen of each mosquito were separated and each part placed in a drop of 1% saline solution on a slide. In the case of *An. gambiae* s.l. the legs were removed and placed in 1.5 ml Eppendorf tubes for molecular identification of the species complex. Each of the body parts were dissected under a dissecting microscope for the presence of *W. bancrofti*. The stored *Mansonia* spp were re-hydrated initially for 5 min in 1% saline solution in 96-well microtitre plates before dissecting as described above.

## Molecular identification of *An. gambiae* species complex

Genomic DNA was extracted from the legs of *An. gambiae* s.l using the method of Collins *et al.*[15]. Identification of the sibling species was done using the method of Scott *et al.*[16] and that of Fanello *et al.* (2002) used to determine the *An. gambiae* s.s M and S molecular forms. [17]

## Molecular identification of *W. bancrofti*

The dried carcass of dissected mosquitoes together with any *W. bancrofti* larvae found on each slide was scraped into a 1.5 ml Eppendorf tube and then homogenized in phosphate buffered saline (PBS). The genomic DNA was extracted using the DNeasy Tissue Kit (QIAGEN Inc., USA) following the manufacturer’s protocol. Polymerase chain reaction to confirm that parasites observed were *W. bancrofti* was then carried out using the method of Ramzy *et al.* (1997) [18].

## Ethical considerations

Ethical approval for both studies was obtained from the Institutional Review Board of the Noguchi Memorial Institute for Medical Research and verbal/written consents were obtained from each local volunteers who participated in the indoor human landing catches (HLC) during July to December which correspond to the period of highest mosquito breeding in Gomoa. Prior consents to conduct pyrethrum spray catches (PSC) in rooms were also obtained from the occupants.

## Results

A total of 825 mosquitoes comprising of 501 (61%) *Anopheles* spp, 239 (29%) *Mansonia* spp, 84 *Culex* spp (10%) and 1 *Aedes* spp from KEEA were studied. Seventy (70) *An. gambiae* s.l. were further identified as *An. gambiae* s.s (n = 58) and *An*. *melas* (n = 3) while nine did not amplify. Thus the ratio of *An. gambiae: An. melas* was approximately 20:1. Twenty-two of the *An. gambiae* s.s were further identified as M (n = 15) and S (n = 7) forms. All the 239 *Mansonia* spp were identified as *M. africana*.

All the 825 mosquitoes from the KEEA were dissected and five *M. africana* and two *An. gambiae* s.s were found to be infective. One *M. africana* was infected with one L_2_ stage of the parasite (Table 1). Thus the estimated infection and infectivity rates for *M. africana* were 2.5% and 2.1% respectively with *An. gambiae* s.l having an infectivity rate of 0.4%. Table 2 shows the details of the infections in individual *Mansonia* species from Sanka where approximately 67% of the total *Mansonia* species from KEEA were obtained. In this community, the five infective *Mansonia* mosquitoes found harboured a total of 26 L_3_s, and three had as many as 6, 7 and 11 L_3_s respectively.

**Table 1:** **The distribution of mosquito species infected and infective with*W. bancrofti*stages observed at KEEA District**

						**Number of infected mosquitoes**		**Number of infective mosquitoes**		**Total number of worms (L1, L2, L3)**	
**Study site**	***Anopheles spp.***	***Aedes spp.***	***Culex spp.***	***Mansonia spp.***	**Total dissected**	***Anopheles spp***	***Mansonia Africana***	***Anopheles spp***	***Mansonia africana***	***Anopheles spp***	***Mansonia africana***
Sanka	33	1	7	160	201	0	5	0	5	0	57
Bandor	60	0	8	20	88	0	0	0	0	0	0
Atabadze	45	0	27	4	76	0	0	0	0	0	0
Anyinase	80	0	30	46	156	1	1	1	0	1	1
Ponkrom	103	0	2	2	107	0	0	0	0	0	0
Epoano	180	0	10	7	197	1	0	1	1	0	1
Total	501	1	84	239	825	2	6	2	6	1	59

**Table 2:** **The number of different larval stages found in the individual + infected*Mansonia africana*at Sanka**

**Village-Code**	**Total number of worms**	**L**_**1**_	**L**_**2**_	**L**_**3**_
Sanka-S/75-19	26	5	10	11
Sanka-S/75-39	19	3	10	6
Sanka-S/75-40	10	2	1	7
Sanka-S/75-38	1			1
Sanka-S/4	1			1

Three hundred and eighty-eight stored *Mansonia* sp. from the Gomoa District collected between 2000–2003 were examined. Of these 144 were identified as *M. africana* and 244 were *M. uniformis*. Eleven *M. africana* were found infected with all stages of the parasite with four of them with infective stages, while seven *M. uniformis* were found infected with two of them with the infective stage. The distribution of the *W. bancrofti* stages among the two *Mansonia* species are also given in Table 3. The infection and infectivity rates for *M. africana* were 7.6% and 2.8% respectively while those for *M. uniformis* were 2.9% and 0.8 respectively.

**Table 3:** **Total number of*Mansonia*sp infected and infective in the stored samples Gomoa district**

***Mansonia sp.***	**Total No dissected**	**No. Infected**	**No. Infective**	**No. of *W. bancrofti *life stages**		
				**L1**	**L2**	**L3**
*M. africana*	144	11	4	1	6	4
*M. uniformis*	244	7	2	3	2	2
Total	388	18	6	4	8	6

The identity of *W. bancrofti* was confirmed by PCR.

## Discussion

In studies on the vectors of lymphatic filariasis in Africa, species of biting mosquitoes collected include the genera *Aedes, CulexAnopheles* and *Mansonia*. Species of these genera are known to transmit lymphatic filariasis in one place or another worldwide. However, in Africa only *Culex* and *Anopheles* are reported as vectors. *Culex* species are important vectors in urban areas of East Africa [19,20], but not in West Africa. A recent study carried out on *Culex* species in Ghana revealed that they are refractory [6]. In West Africa, including Ghana, *Anopheles* species have been reported as the only vectors of *W. bancrofti*[1-3], although there has been an earlier report of L_3_ of *W. bancrofti* in *M. uniformis* from Boké, Guinea [7]. Even though *Mansonia* species are known vectors of filariasis in some endemic countries in Asia they had not been considered as vectors in Africa. A recent study however, revealed that infective *M. uniformis* was possible in laboratory experiments but this was not the case in the wild [21]. Therefore, this is the first report of infection of all stages of *W. bancrofti* in *Mansonia* species in Ghana and the only one in West Africa since 1958.

The global strategy for the elimination of LF is based on the vector –parasite relationships of facilitation and limitation. *Anopheles* vectors of *W. bancrofti* have been reported to exhibit facilitation and hence elimination is feasible through MDA alone where they are the vectors. Thus West Africa is among the areas where LF elimination through MDA is expected to progress without any hitch. However, in some sites in Ghana and Burkina Faso, the prevalence of infection is not what is expected after 5 to 8 years of annual MDA. For example in the KEEA district a study conducted after 6 annual MDA showed a prevalence of 2.1% (23/1107) in some neighbouring communities to the study sites (D. Boakye unpublished report). There have been various explanations for this apparent residual infection and non-compliance by infected people to take the drugs have been postulated as was found in Haiti [22]. Others have hypothesized that on-going transmission could account for the prevalence observed and that not all *Anopheles* species may exhibit facilitation [23]. A recent study by Amuzu *et al.* at the Gomoa District sites showed that *An. melas* may be exhibiting limitation and could be responsible for transmission at low level parasitaemia [24]. It has also been suggested that even if vectors pick up parasites after MDA, the number of parasites ingested could be too low to sustain effective transmission. In this study, we found an individual infective *Mansonia* species that harboured up to 11 L_3_ larvae. This indicates that an infective bite could transmit many parasites that could set up an active infection. Hence, transmission could be sustained even if at a low level. Relatively, therefore, it does appear that *Mansonia* species could be more important compared to *An. gambiae* species especially at low level microfilaraemia.

Previous studies in some parts of West Africa and in Accra Ghana as summarized by Laurence, shows that *Mansonia africana* was more widely distributed than *Mansonia uniformis* and that their distribution does not completely coincide [25]. We found this to be the case since both study sites have similar ecology and housing structure but *Mansonia uniformis* was localized in only one study area. Even though sampling at both sites was carried out in the rainy season, the study was limited by the fact that it was not carried out at the same time. The sampling methods used at the study sites were also different and these could have been a contributing factor for the differences in the distribution of the two species.

There is currently an ethical debate on the use of the human landing collection (HLC) for sampling vector species. However, HLC provides accurate estimates of transmission indices due to anthropophilic vectors, which are difficult to obtain from other collection methods. For example, mosquitoes obtained from other methods such as the indoor residual spraying collections require analysis of bloodmeals to indicate the level of anthropophily (human biting index). In the current study, the mosquitoes processed from the Gomoa district were part of sampling to accurately determine the impact of MDA on transmission of *W. bancrofti* hence the use of HLC.

## Conclusions

The observation from this study that *M. uniformis* and *M. africana* are vectors in the study sites show that the transmission system could be more complex than expected in some areas in West Africa. *Mansonia* species are seemingly important vectors, which have not been factored in the elimination strategy in the sub-region and therefore could have critical implications. Their distribution, biology and vectorial importance should be studied as well as the need to study local vectorial systems so as to complement MDA with vector control where necessary for the goals of GPELF to be achieved.

## Competing interests

The authors declare that they have no competing interests.
